# Late Presentation With HIV in Africa: Phenotypes, Risk, and Risk Stratification in the REALITY Trial

**DOI:** 10.1093/cid/cix1142

**Published:** 2018-03-04

**Authors:** Abraham Siika, Leanne McCabe, Mutsa Bwakura-Dangarembizi, Cissy Kityo, Jane Mallewa, Jay Berkley, Kath Maitland, Anna Griffiths, Keith Baleeta, Shepherd Mudzingwa, James Abach, Kusum Nathoo, Margaret J Thomason, Andrew J Prendergast, Ann Sarah Walker, Diana M Gibb, REALITY Trial Team, P Mugyenyi, C Kityo, V Musiime, P Wavamunno, E Nambi, P Ocitti, M Ndigendawani, M Kemigisa, J Acen, D Olebo, G Mpamize, A Amone, D Okweny, A Mbonye, F Nambaziira, A Rweyora, M Kangah, V Kabaswahili, J Abach, G Abongomera, J Omongin, I Aciro, A Philliam, B Arach, E Ocung, G Amone, P Miles, C Adong, C Tumsuiime, P Kidega, B Otto, F Apio, K Baleeta, A Mukuye, M Abwola, F Ssennono, D Baliruno, S Tuhirwe, R Namisi, F Kigongo, D Kikyonkyo, F Mushahara, D Okweny, J Tusiime, A Musiime, A Nankya, D Atwongyeire, S Sirikye, S Mula, N Noowe, A Lugemwa, M Kasozi, S Mwebe, L Atwine, T Senkindu, T Natuhurira, C Katemba, E Ninsiima, M Acaku, J Kyomuhangi, R Ankunda, D Tukwasibwe, L Ayesiga, J Hakim, K Nathoo, M Bwakura-Dangarembizi, A Reid, E Chidziva, T Mhute, GC Tinago, J Bhiri, S Mudzingwa, M Phiri, J Steamer, R Nhema, C Warambwa, G Musoro, S Mutsai, B Nemasango, C Moyo, S Chitongo, K Rashirai, S Vhembo, B Mlambo, S Nkomani, B Ndemera, M Willard, C Berejena, Y Musodza, P Matiza, B Mudenge, V Guti, A Etyang, C Agutu, J Berkley, K Maitland, P Njuguna, S Mwaringa, T Etyang, K Awuondo, S Wale, J Shangala, J Kithunga, S Mwarumba, S Said Maitha, R Mutai, M Lozi Lewa, G Mwambingu, A Mwanzu, C Kalama, H Latham, J Shikuku, A Fondo, A Njogu, C Khadenge, B Mwakisha, A Siika, K Wools-Kaloustian, W Nyandiko, P Cheruiyot, A Sudoi, S Wachira, B Meli, M Karoney, A Nzioka, M Tanui, M Mokaya, W Ekiru, C Mboya, D Mwimali, C Mengich, J Choge, W Injera, K Njenga, S Cherutich, M Anyango Orido, G Omondi Lwande, P Rutto, A Mudogo, I Kutto, A Shali, L Jaika, H Jerotich, M Pierre, J Mallewa, S Kaunda, J Van Oosterhout, B O’Hare, R Heydermann, C Gonzalez, N Dzabala, C Kelly, B Denis, G Selemani, L Nyondo Mipando, E Chirwa, P Banda, L Mvula, H Msuku, M Ziwoya, Y Manda, S Nicholas, C Masesa, T Mwalukomo, L Makhaza, I Sheha, J Bwanali, M Limbuni, D Gibb, M Thomason, AS Walker, S Pett, A Szubert, A Griffiths, H Wilkes, C Rajapakse, M Spyer, A Prendergast, N Klein, N Van Looy, E Little, K Fairbrother, F Cowan, J Seeley, S Bernays, R Kawuma, Z Mupambireyi, F Kyomuhendo, S Nakalanzi, J Peshu, S Ndaa, J Chabuka, N Mkandawire, L Matandika, C Kapuya, I Weller, E Malianga, C Mwansambo, F Miiro, P Elyanu, E Bukusi, E Katabira, O Mugurungi, D Gibb, J Hakim, A Etyang, P Mugyenyi, J Mallewa, T Peto, P Musoke, J Matenga, S Phiri, H Lyall, V Johnston, F Fitzgerald, F Post, F Ssali, A Prendergast, A Arenas-Pinto, A Turkova, A Bamford

**Affiliations:** 1 Moi University School of Medicine, Eldoret, Kenya; 2 Medical Research Council Clinical Trials Unit at University College London, United Kingdom; 3 University of Zimbabwe Clinical Research Centre, Harare; 4 Joint Clinical Research Centre, Kampala, Uganda; 5 Department/College of Medicine and Malawi-Liverpool–Wellcome Trust Clinical Research Programme, Blantyre; 6 Kenya Medical Research Institute–Wellcome Trust Research Programme, Kilifi; 7 Joint Clinical Research Centre, Mbale; 8 Joint Clinical Research Centre, Gulu, Uganda; 9 Queen Mary University of London, United Kingdom

**Keywords:** HIV, Africa, immunosuppression, mortality

## Abstract

**Background:**

Severely immunocompromised human immunodeficiency virus (HIV)–infected individuals have high mortality shortly after starting antiretroviral therapy (ART). We investigated predictors of early mortality and “late presenter” phenotypes.

**Methods:**

The Reduction of EArly MortaLITY (REALITY) trial enrolled ART-naive adults and children ≥5 years of age with CD4 counts <100 cells/µL initiating ART in Uganda, Zimbabwe, Malawi, and Kenya. Baseline predictors of mortality through 48 weeks were identified using Cox regression with backwards elimination (exit *P* > .1).

**Results:**

Among 1711 included participants, 203 (12%) died. Mortality was independently higher with older age; lower CD4 count, albumin, hemoglobin, and grip strength; presence of World Health Organization stage 3/4 weight loss, fever, or vomiting; and problems with mobility or self-care at baseline (all *P* < .04). Receiving enhanced antimicrobial prophylaxis independently reduced mortality (*P* = .02). Of five late-presenter phenotypes, Group 1 (n = 355) had highest mortality (25%; median CD4 count, 28 cells/µL), with high symptom burden, weight loss, poor mobility, and low albumin and hemoglobin. Group 2 (n = 394; 11% mortality; 43 cells/µL) also had weight loss, with high white cell, platelet, and neutrophil counts suggesting underlying inflammation/infection. Group 3 (n = 218; 10% mortality) had low CD4 counts (27 cells/µL), but low symptom burden and maintained fat mass. The remaining groups had 4%–6% mortality.

**Conclusions:**

Clinical and laboratory features identified groups with highest mortality following ART initiation. A screening tool could identify patients with low CD4 counts for prioritizing same-day ART initiation, enhanced prophylaxis, and intensive follow-up.

**Clinical Trials Registration:**

ISRCTN43622374.

World Health Organization (WHO) guidelines recommend universal antiretroviral therapy (ART) irrespective of CD4 count [1–3]. However, in sub-Saharan Africa, 20%–25% of human immunodeficiency virus (HIV)–infected individuals still present for care with severe immunosuppression (CD4 count <100 cells/µL) [4], of whom approximately 10% die within 3 months of ART initiation [5–8]. Early mortality is markedly higher in adults and children with low CD4 count or lower body mass index (BMI), even above levels indicating severe malnutrition [7–9], or with low hemoglobin level [8].

A recent analysis of 850 HIV-infected adults/adolescents initiating ART with CD4 count <50 cells/µL, randomized to receive empiric tuberculosis treatment or isoniazid preventive therapy, identified higher mortality in those with lower CD4 cell count, albumin, and hemoglobin; higher white blood cell (WBC) count or neutrophil percentage; or lymphadenopathy [10]. Given the substantial numbers presenting with low CD4 cell count and their high mortality shortly after ART initiation, additional studies in “late presenters” are important to identify their key characteristics, particularly if baseline CD4 count testing is not always available, to identify them earlier at healthcare facilities and prioritize them for additional interventions.

We therefore investigated predictors of mortality in the first 48 weeks on ART, and characterized different patterns of late presentation in adults and older children enrolled in the Reduction in EArly MortaLITY (REALITY) trial (ISRCTN43622374).

## METHODS

REALITY recruited previously untreated adults and children aged ≥5 years with a CD4 count <100 cells/µL from 8 centers in Kenya, Malawi, Uganda, and Zimbabwe [11]. Pregnancy or breastfeeding were trial exclusion criteria. Participants were randomized 1:1 to each of 12 weeks of raltegravir vs no raltegravir; enhanced prophylaxis vs standard prophylaxis (cotrimoxazole); and 12 weeks of ready-to-use supplementary food vs standard nutritional support, together with WHO-recommended combination ART. Enhanced prophylaxis consisted of cotrimoxazole plus isoniazid, pyridoxine, and fluconazole for 12 weeks, azithromycin for 5 days, and a single dose of albendazole [11]. Adults and children’s guardians gave written informed consent; older children gave additional assent following national guidelines. The trial was approved by ethics committees in Zimbabwe, Uganda, Malawi, Kenya, and the United Kingdom.

At screening and enrollment, patients underwent a physical examination including height, weight, mid-upper arm circumference (MUAC), body composition assessment by bioimpedance analysis (TANITA BC-420MA), and assessment for WHO stage 3/4 events, including tuberculosis symptom screen and diagnostic tests as indicated. Concomitant medications, including reason for prescription, were also recorded at both visits. At enrollment, blood pressure and grip strength (Takei 5401 Hand Grip Dynamometer) were measured, and participants were administered a symptom checklist and EuroQol 5 Dimensions (EQ-5D) questionnaire. Full blood count, tests of renal and liver function, and lymphocyte subsets were assayed at screening and enrollment in real time and samples were stored at enrollment for retrospective HIV viral load (VL) assays.

### Statistical Analysis

Baseline demographics, laboratory results, physical measurements, and EQ-5D scores were all considered as predictors of mortality during the first 48 weeks on ART, after which participants exited the trial (Table 1; Supplementary Table 1; details in Supplementary Methods). Factors were selected for the final Cox proportional hazards model using backwards elimination (exit *P* > .1 to build an explanatory model, reporting only factors with *P* < .05) and incorporating any significant nonlinearity (Stata mfp). Clinical center (reflecting clinical management and access to diagnostic facilities) was included in all models. Models were restricted to complete cases. Different patterns of late presenters were identified using hierarchical cluster analysis determining the number of clusters using the Duda-Hart stopping rule [12] (details are shown in the Supplementary Methods). Analysis was performed using Stata software version 14.2.

**Table 1.** Characteristics at Antiretroviral Therapy Initiation

Characteristic	Died Before 48 wk (n = 225)	Last Seen Alive (n = 1580)	All Participants (N = 1805)	Univariable *P* Value^a^
Male sex	109 (48)	852 (54)	961 (53)	.12
Age, y	37 (30–44)	36 (29–41)	36 (29–42)	.005
Age 5–17 y	4 (2)	68 (4)	72 (4)	
Center				<.001
Blantyre, Malawi	25 (11)	230 (15)	255 (14)	
Harare, Zimbabwe	51 (23)	518 (33)	569 (32)	
Kilifi, Kenya	29 (13)	114 (7)	143 (8)	
Eldoret, Kenya	14 (6)	194 (12)	208 (12)	
Fort Portal, Uganda	28 (12)	114 (7)	142 (8)	
Mbale, Uganda	25 (11)	94 (6)	119 (7)	
Mbarara, Uganda	26 (12)	203 (13)	229 (13)	
Gulu, Uganda	27 (12)	113 (7)	140 (8)	
Smoking status (n = 1795)				.86
Never	180 (80)	1269 (81)	1449 (81)	
Ever	45 (20)	301 (19)	346 (19)	
Blood pressure (systolic/diastolic), mm Hg (n = 1767)	102/69 (93/60–110/75)	109/70 (100/63–119/78)	108/70 (99/63–118/77)	SBP <.001; DBP .006
BMI, kg/m^2^ (n = 1797)	18.0 (16.2–19.8)	19.3 (17.4–21.6)	19.2 (17.2–21.4)	<.001
MUAC, cm (n = 1804)	22.6 (20.2–24.7)	24.0 (22.0–26.3)	24.0 (21.8–26.0)	<.001
Grip strength, kg (n = 1772)	20.0 (15.3–25.6)	25.1 (19.9–31.4)	24.5 (19.3–31.0)	<.001
Fat mass, kg (n = 1743)	4.6 (1.8–8.9)	6.7 (3.8–12.5)	6.5 (3.6–12.2)	<.001
Fat-free mass, kg (n = 1743)	40.9 (37.2–47.3)	43.9 (39.4–49.7)	43.5 (39.0–49.4)	<.001
HIV VL, copies/mL (n = 1790)	332260 (132020–1000000)	240000 (93000–554010)	249765 (95630–606310)	
Log_10_ HIV VL (n = 1790)	5.5 (5.1–6.0)	5.4 (5.0–5.7)	5.4 (5.0–5.8)	<.001
CD4 count, cells/µL	25 (10–51)	38 (17–64)	37 (16–63)	<.001
Hemoglobin, g/dL (n = 1802)	10.0 (8.4–11.6)	11.4 (9.7–12.8)	11.2 (9.6–12.7)	<.001
WBC count, 10^9^/L (n = 1802)	3.9 (2.9–5.4)	3.5 (2.7–4.6)	3.5 (2.7–4.7)	<.001
Platelets, 10^9^/L (n = 1801)	260 (189–341)	251 (182–343)	252 (183–343)	.88
Neutrophils, 10^9^/L (n = 1785)	1.99 (1.38–3.07)	1.69 (1.15–2.50)	1.72 (1.17–2.58)	<.001
Albumin, g/L (n = 1755)	30 (26–35)	35 (31–40)	35 (30–40)	<.001
eGFR^b^, mL/min (n = 1793)	85.3 (60.4–111.3)	98.8 (79.3–122.4)	97.3 (77.3–121.1)	<.001
Hospitalized at randomization	11 (5)	11 (1)	22 (1)	<.001
Previous healthcare contact^c^	23 (10)	140 (9)	163 (9)	.50
Patient-reported symptoms at enrollment				
Fever	70 (31)	170 (11)	240 (13)	<.001
Weight loss	147 (65)	759 (48)	906 (50)	<.001
Difficulty walking	86 (38)	148 (9)	234 (13)	<.001
Rash	34 (15)	291 (18)	325 (18)	.22
Numbness	60 (27)	293 (19)	353 (20)	.004
Abdominal ache	39 (17)	157 (10)	196 (11)	.001
Sore mouth	34 (15)	148 (9)	182 (10)	.004
Vomiting	39 (17)	75 (5)	114 (6)	<.001
Physician-assessed illnesses				
Current wasting/severe weight loss (WHO stage 3/4)	64 (28)	263 (17)	327 (18)	<.001
Current TB (all) (WHO stage 3/4)	48 (21)	223 (14)	271 (15)	.003
Current *Candida* infection (WHO stage 3/4)	17 (8)	82 (5)	99 (5)	.10
EQ-5D				
Mobility (n = 1791)				<.001
No problems walking	102 (46)	1297 (83)	1399 (78)	
Some problems walking	103 (47)	261 (17)	364 (20)	
Confined to bed	16 (7)	12 (1)	28 (2)	
Self-care (n = 1791)				<.001
No problems washing/dressing	110 (50)	1318 (84)	1428 (80)	
Some problems washing/dressing	74 (33)	212 (14)	286 (16)	
Unable to wash/dress myself	37 (17)	40 (3)	77 (4)	
Usual activities (n = 1791)				<.001
No problems with usual activities	91 (41)	1160 (74)	1251 (70)	
Some problems with usual activities	80 (36)	319 (20)	399 (22)	
Unable to do usual activities	50 (23)	91 (6)	141 (8)	
Pain/discomfort (n = 1791)				<.001
No pain/discomfort	93 (42)	1009 (64)	1102 (62)	
Moderate pain/discomfort	116 (52)	517 (33)	633 (35)	
Extreme pain/discomfort	12 (5)	44 (2)	56 (3)	
Anxiety (n = 1791)				<.001
Not anxious/depressed	121 (55)	1191 (76)	1312 (73)	
Moderately anxious/depressed	89 (40)	348 (22)	437 (24)	
Extremely anxious/depressed	11 (5)	31 (2)	42 (2)	
WHO stage				<.001
1	22 (10)	278 (18)	300 (17)	
2	44 (19)	510 (32)	554 (31)	
3	105 (47)	586 (37)	691 (38)	
4	54 (24)	206 (13)	260 (14)	
Randomized to enhanced prophylaxis	98 (44)	808 (51)	906 (50)	.03
Randomized to adjunctive raltegravir	110 (49)	792 (50)	902 (50)	.77
Randomized to adjunctive ready-to-use food	111 (49)	786 (50)	897 (50)	.94

Data are presented as No. (%) or median (interquartile range).

Abbreviations: BMI, body mass index; DBP, diastolic blood pressure; eGFR, estimated glomerular filtration rate; EQ-5D, EuroQol 5 Dimensions (score); HIV, human immunodeficiency virus; MUAC, mid-upper arm circumference; SBP, systolic blood pressure; TB, tuberculosis; VL, viral load; WBC, white blood cell; WHO, World Health Organization.

^a^From Cox model.

^b^Estimated using Cockcroft-Gault formula.

^c^Chronic health conditions or prescribed medications >14 days prior to screening visit.

## RESULTS

A total of 1805 participants were recruited, 40 (2%) aged <13 years, all previously untreated and with CD4 count <100 cells/µL at screening following the trial design, and hence considered late presenters. Two hundred twenty-five (12%) died before 48 weeks, a median of 8 weeks (interquartile range [IQR], 3–18 weeks) after ART initiation. Only 56 (3%) participants were lost to follow-up. Although 1674 (93%) participants reported an HIV test prior to trial screening, the time since this test was very short (median, 14 days; IQR, 6–39 days). Participants who died were slightly older (median, 37 vs 36 years; *P* = .005; Table 1) and had significantly lower baseline CD4 counts (median, 25 vs 38 cells/µL; *P* < .001). Excepting platelets, all other laboratory tests differed significantly in those who died (*P* < .01): Hemoglobin, albumin, and estimated glomerular filtration rate (Cockcroft-Gault formula) were all lower in those who died whereas HIV VL, WBC count, and neutrophils were higher. All baseline physical measurements and BMI were significantly lower in those who subsequently died (*P* < .001), particularly grip strength (median, 20.0 vs 25.1 kg) and fat mass (median, 4.6 vs 6.7 kg). All symptoms except rash were reported more at enrollment in those who subsequently died (*P* < .01), and they were significantly more likely to suffer from WHO stage 3/4 weight loss and tuberculosis (*P* < .01) at enrollment. Patients who died were significantly more likely to report moderate or extreme problems for EQ-5D (*P* < .001).

Final multivariable models included 1711 participants (26 aged <13 years), of whom 203 (12%) died. Mortality was independently higher in those who were older (*P* = .002), with lower CD4 counts (*P* < .001), lower albumin (*P* = .001), lower hemoglobin (*P* = .01), and weaker grip strength (*P* = .03); those in whom physicians reported WHO stage 3/4 weight loss (*P* = .04); and in those patients reporting fever (*P* = .001), vomiting (*P* = .02), some problems with mobility (*P* = .005), and inability to wash or dress themselves (*P* = .003) at baseline (Table 2). Receiving enhanced prophylaxis independently reduced mortality (*P* = .02) (as overall in [11]), but receiving raltegravir (*P* = .60) and ready-to-use supplementary food (*P* = .37) did not, and were not included in the final model. The area under the receiver operating curve was 80% (95% confidence interval, 76%–83%), supporting good model discrimination. CD8 cell count was not considered in this multivariable model as this would have excluded an additional 108 (6%) participants; however, CD8 cell count was not associated with mortality after adjusting for other factors in Table 2 (*P* = .41).

**Table 2.** Independent Predictors of Mortality in the First 48 Weeks on Antiretroviral Therapy Initiated With CD4 Count <100 Cells/µL

Factor	Univariable Effects of Factors Selected for Multivariable Model in REALITY Trial Participants			Final Multivariable Model in REALITY Trial Participants			Multivariable Model From Bisson et al [10], Fitted to REALITY Trial Participants		
	HR	(95% CI)	*P* Value	HR	(95% CI)	*P* Value	HR	(95% CI)	*P* Value
Age (per 5 y older)	1.09	(1.03–1.16)	.005	1.12	(1.04–1.20)	.002	1.13	(1.06–1.21)	<.001
CD4 (per 10 cells/µL higher)	0.89	(.85–.94)	<.001	0.90	(.85–.95)	<.001	0.87	(.82–.92)	<.001
Hemoglobin (per g/dL higher)	0.77	(.72–.82)	<.001	0.90	(.83–.99)	.01	0.87	(.80–.94)	<.001
Albumin (per g/L higher)	0.92	(.90–.93)	<.001	0.96	(.94–.98)	.001	0.94	(.92–.97)	<.001
WBC count (per 10^9^/L higher)	1.17	(1.08–1.26)	<.001	1.08	(1.00–1.17)	.054	1.16	(1.06–1.25)	.001
Grip strength (per kg higher)	0.93	(.91–.95)	<.001	0.98	(.96–1.00)	.03	…		
Previous healthcare contact	1.16	(.75–1.79)	.50	0.63	(.39–1.03)	.067	…		
WHO stage 3/4 weight loss	1.90	(1.42–2.53)	<.001	1.43	(1.01–2.03)	.04	…		
Patient-reported fever	3.37	(2.54–4.47)	<.001	1.67	(1.19–2.35)	.003	…		
Patient-reported vomiting	3.72	(2.64–5.26)	<.001	1.55	(1.00–2.39)	.048	…		
EQ-5D Mobility: some problems vs no problems	4.42	(3.36–5.81)	<.001	1.88	(1.21–2.93)	.005	…		
Confined to bed vs no problems	11.87	(7.00–20.12)	<.001	2.47	(1.00–6.08)	.050	…		
EQ-5D Self-care: some problems vs no problems	3.73	(2.78–5.02)	<.001	1.32	(.82–2.13)	.26	…		
Unable to wash/dress vs no problems	8.59	(5.91–12.47)	<.001	2.78	(1.42–5.43)	.003	…		
Enhanced prophylaxis	0.75	(.57–.97)	.03	0.72	(.54–.96)	.02	…		
Sex (female vs male)	1.23	(.95–1.60)	.12	…			1.19	(.89–1.60)	.23
BMI <18.5 vs >25 kg/m^2^	2.41	(1.30–4.48)	.005	…			0.90	(.52–1.55)	.70
BMI 18.5–25 vs >25 kg/m^2^	1.38	(.74–2.58)	.32	…			0.70	(.41–1.19)	.19
HIV VL (per log_10_ higher)	1.52	(1.22–1.89)	<.001	…			1.37	(1.09–1.74)	.008
Neutrophil % (per 5% higher)	1.07	(1.02–1.12)	.003	…			0.99	(.95–1.04)	.64

Final multivariable model in REALITY trial participants also adjusted for center. Backwards elimination (exit *P* < .1) on factors in Table 1, refitting final model to all observations with complete data for chosen factors. Factors with *P* > .05 are included to adjust for confounding. All continuous factors were selected in final models with linear effects, ie, each unit increase had the same impact on mortality at all levels of the factor.

Abbreviations: BMI, body mass index; CI, confidence interval; EQ-5D, EuroQol 5 Dimensions (score); HIV, human immunodeficiency virus; HR, hazard ratio; REALITY, Reduction in EArly MortaLITY (trial); VL, viral load; WBC, white blood cell; WHO, World Health Organization.

**Figure 1.**
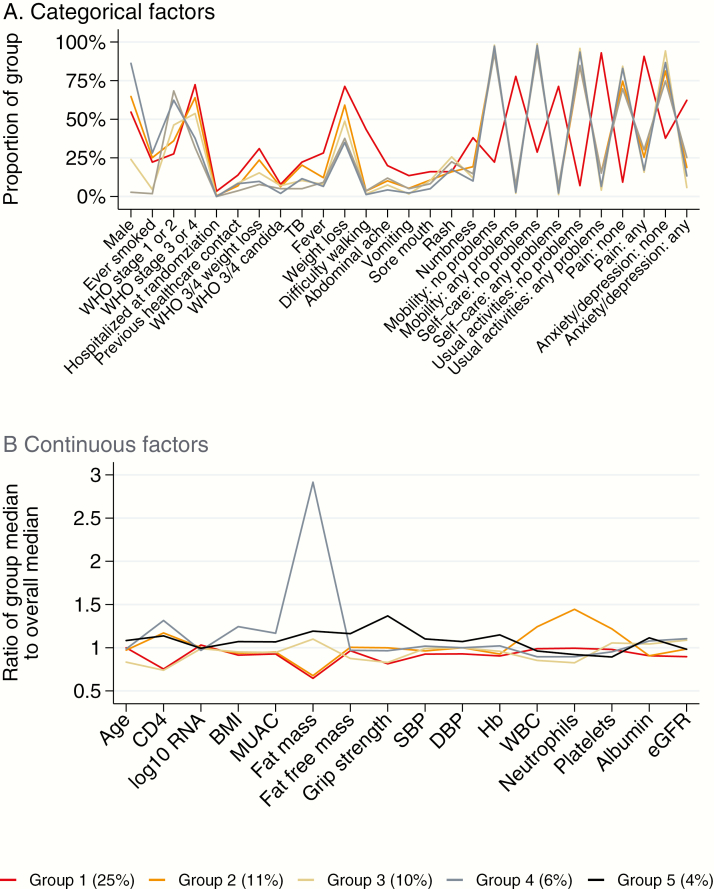
Summary of characteristics of different patterns of late presenters. *A*, Categorical factors. *B*, Continuous factors. Percentage of participants who died in each group is indicated in parentheses at bottom. See Supplementary Table 2 for full details of characteristics in each group. Abbreviations: BMI, body mass index; DBP, diastolic blood pressure; eGFR, estimated glomerular filtration rate; Hb, hemoglobin; MUAC, mid-upper arm circumference; SBP, systolic blood pressure; TB, tuberculosis; WBC, white blood cell; WHO, World Health Organization.

We then considered whether the risk factors mentioned above clustered in specific groups of patients, that is, whether late presenters could be phenotyped according to their pre-ART characteristics. Five groups of the 1607 participants with complete data for all factors in Table 1 were identified through clustering. Group 1 had the highest mortality (25% [87/355]; 18% by week 12) and were characterized by high burden of problems reported on the EQ-5D, particularly for mobility (78% vs 20% overall), self-care (71% vs 18%), and usual activities (93% vs 29%; Supplementary Table 2; represented visually in Figure 1). Excluding rash, they also had the highest burden of symptoms and illness, especially weight loss, difficulty walking, and poor appetite.

Other groups had significantly lower, but still substantial, mortality. Group 2 (n = 394; 11% [45] deaths; 7% by week 12) had high levels of WBCs, platelets, and neutrophils, despite lower levels of reported fever (12%) than group 1 (28%). Only 122 (31%) had an infection reported at enrollment, suggesting they may have had underlying infections or inflammation. This group also had substantial weight loss and low fat mass, and low BMI, albumin, and hemoglobin, similar to group 1. However, median CD4 cell count was higher in group 2 vs group 1 (43 vs 28 cells/µL) as was grip strength (median, 24.5 vs 20.0 kg).

Group 3 (n = 242; 10% [25] deaths; 5% by week 12) were mainly (nonpregnant, nonbreastfeeding) females (76%) and younger (median, 30 vs 36 years overall). They had low CD4 counts (median, 27 cells/µL), WBCs, neutrophils, hemoglobin, BMI, MUAC, and grip strength, but despite this had low burden of symptoms/illnesses and maintained a reasonable fat mass and higher albumin. Group 4 (n = 218; 6% [12] deaths) was also mostly female (97%), but differed from group 3 in being older (median, 36 vs 30 years) with higher CD4 cell counts (median, 48 vs 27 cells/µL) and much higher fat mass (median, 18.9 vs 7.1 kg in group 3; 6.6 kg overall), with correspondingly higher BMI and MUAC. Group 5 (n = 398; 4% [15] deaths) was predominately male (86%) and older (median, 39 years) with higher fat mass, fat-free mass, and grip strength, and a median CD4 count of 42 cells/µL.

Cluster significantly predicted mortality univariably (*P* < .001), but not after adjusting for the factors shown in Table 2 (*P* = .18), as expected since the clusters were predominantly differentiated by clinical factors (Supplementary Table 2). There was no evidence that the effect of enhanced prophylaxis on mortality varied across the clusters (interaction *P* = .32), nor did the effect of adjunctive raltegravir (*P* = .20) or supplementary food (*P* = .66).

## DISCUSSION

Using data from a large African trial in which 12% of HIV-infected adults and older children with a CD4 count <100 cells/µL died during the 48 weeks following ART initiation, we confirm the importance of weight loss, clinical symptoms, and activities of daily living as predictors of mortality, independent of low CD4 cell counts. Low hemoglobin and albumin were the only laboratory prognostic markers of early mortality in these late presenters, in addition to CD4 cell count, as in another smaller recent study [10]. However, we identified a constellation of clinical symptoms and other laboratory markers which characterized subgroups of patients at even higher mortality risk.

As we collected data on a large number of clinical and laboratory parameters (Table 1; Supplementary Table 1), we were able to identify a more detailed and slightly different set of clinical predictors to a recent analysis of a smaller trial with lower mortality (7%) [10]. Interestingly, high HIV VL and WBC count, but not neutrophil percentage, were independent predictors of mortality in REALITY participants when fitting the prognostic model from this previous study (Table 2). However, other clinical factors were more predictive than these laboratory parameters in REALITY participants. In particular, once problems with mobility were included as a prognostic factor, there was no additional association between HIV VL and mortality, suggesting that mobility may be a broad physical marker for the detrimental impact of ongoing viral replication. There was a marginal association between WBC and mortality in our final model, supporting infection and/or inflammation playing a role in early increased mortality risk. These clinical factors are not part of traditional WHO staging, which is based on defined illnesses, but may be helpful for triaging patients at high risk of early mortality on ART for more intensive monitoring and/or support, particularly in lower-level health facilities when access to laboratory tests is limited.

We previously showed that a package of enhanced prophylaxis reduced mortality by 27% during the first 24 weeks of ART in this population with low CD4 counts [11]. Here, we found no evidence that any group benefited more from enhanced prophylaxis, although power was limited to detect greater benefits in specific subgroups. It is therefore important that CD4 monitoring is available in health facilities to identify late presenters, particularly as almost half of participants in this trial had minimal clinical symptoms, in particular the predominantly young female group 3 with very low CD4 cell counts. Simple questions about fever, vomiting, weight loss, and activities of daily living (in the EQ-5D) identified those at particularly high risk of mortality who should be prioritized for immediate treatment (eg, same-day ART initiation), enhanced prophylaxis, and more intensive follow-up.

Rather than late presenters being a homogenous group, we identified 5 phenotypes, with several prognostic factors varying substantially across groups, as did mortality. One group had very high mortality (25%), with high levels of wasting and clinical symptoms, and low CD4 count (median, 28 cells/µL), hemoglobin, and albumin, reflecting “traditional” HIV disease, but very high impairment of activities of daily living. There was no evidence that adjunctive raltegravir or supplementary food had any beneficial effects in this subgroup at particularly high mortality risk. Despite similarly low CD4 cell count (median, 27 cells/µL), hemoglobin, and BMI, another group of predominantly younger women without clinical symptoms had significantly lower mortality (although this was still 10%). Another group with 11% mortality had high WBCs, platelets, and neutrophils, suggesting possible underlying infection or chronic inflammation. Future substudies will evaluate how inflammatory markers such as C-reactive protein and interleukin 6 vary between groups, as baseline inflammation independently predicts mortality in adults and children [13–15]. We speculate that groups 1 and 2 have the highest inflammation, which can inhibit protein synthesis and bone marrow activity, leading to low albumin and hemoglobin, and drive cachexia through loss of lean mass. In contrast, the other 3 groups had managed to maintain their weight and body composition (fat mass in particular), despite substantial CD4 impairment, suggesting lower levels of inflammation. Among patients with similar CD4 cell counts, heightened inflammation may drive higher mortality in a subgroup, who might be identified through weight loss, anemia, and hypoalbuminemia.

In summary, a substantial proportion of HIV-infected individuals continue to present for care with advanced immunosuppression. CD4 counts are important to identify those who would benefit from additional interventions [16] with ART, such as enhanced antimicrobial prophylaxis [11], particularly as many present to care without clinical symptoms. Here, we show that clinical and laboratory characteristics could identify groups with highest risk of mortality following ART initiation, despite similarly low CD4 cell counts. Screening patients with low CD4 counts at baseline for significant weight loss, a small cluster of important symptoms (eg, fever and vomiting), impairment of activities of daily living, and a simple assessment of grip strength might identify those at highest risk of death. At higher-level health facilities, additional laboratory tests (albumin and hemoglobin) would also help to stratify high-risk individuals. A screening tool appropriate to the level of facility could therefore help identify which patients with low CD4 cell counts should be prioritized, for example, for same-day ART and enhanced prophylaxis initiation or more intensive follow-up.

## Supplementary Data

Supplementary materials are available at *Clinical Infectious Diseases* online. Consisting of data provided by the authors to benefit the reader, the posted materials are not copyedited and are the sole responsibility of the authors, so questions or comments should be addressed to the corresponding author.

Supplemental Data

## Notes

***Acknowledgments.*** We thank all of the participants and staff from all the centers participating in the REALITY trial. We particularly thank Professor Andrew Prentice for helpful advice on body composition and functional assessment tools.

***Financial support.*** REALITY was funded by the Joint Global Health Trials Scheme (JGHTS) of the UK Department for International Development, the Wellcome Trust, and Medical Research Council (MRC) (grant number G1100693). Additional funding support was provided by the PENTA Foundation and core support to the MRC Clinical Trials Unit at University College London (grant numbers MC_UU_12023/23 and MC_UU_12023/26). Cipla Ltd, Gilead Sciences, ViiV Healthcare/GlaxoSmithKline, and Merck Sharp & Dohme donated drugs for REALITY, and ready-to-use supplementary food was purchased from Valid International. A. J. P. is funded by the Wellcome Trust (grant number 108065/Z/15/Z). J. A. B. is funded by the JGHTS (grant number MR/M007367/1). The Malawi-Liverpool–Wellcome Trust Clinical Research Programme, University of Malawi College of Medicine (grant number 101113/Z/13/Z) and the Kenya Medical Research Institute (KEMRI)/Wellcome Trust Research Programme, Kilifi (grant number 203077/Z/16/Z) are supported by strategic awards from the Wellcome Trust, United Kingdom. Permission to publish was granted by the Director of KEMRI. This supplement was supported by funds from the Bill & Melinda Gates Foundation.

***Supplement sponsorship.*** This article appears as part of the supplement “Advanced HIV Disease,” sponsored by the World Health Organization.

***Potential conflicts of interest.*** All authors: No reported conflicts of interest. All authors have submitted the ICMJE Form for Disclosure of Potential Conflicts of Interest. Conflicts that the editors consider relevant to the content of the manuscript have been disclosed.

## APPENDIX

### REALITY Trial Group

*Participating centers*: Joint Clinical Research Centre (JCRC), Kampala, Uganda (coordinating center for Uganda): P Mugyenyi, C Kityo, V Musiime, P Wavamunno, E Nambi, P Ocitti, M Ndigendawani. JCRC, Fort Portal, Uganda: S Kabahenda, M Kemigisa, J Acen, D Olebo, G Mpamize, A Amone, D Okweny, A Mbonye, F Nambaziira, A Rweyora, M Kangah and V Kabaswahili. JCRC, Gulu, Uganda: J Abach, G Abongomera, J Omongin, I Aciro, A Philliam, B Arach, E Ocung, G Amone, P Miles, C Adong, C Tumsuiime, P Kidega, B Otto, F Apio. JCRC, Mbale, Uganda: K Baleeta, A Mukuye, M Abwola, F Ssennono, D Baliruno, S Tuhirwe, R Namisi, F Kigongo, D Kikyonkyo, F Mushahara, D Okweny, J Tusiime, A Musiime, A Nankya, D Atwongyeire, S Sirikye, S Mula, N Noowe. JCRC, Mbarara, Uganda: A Lugemwa, M Kasozi, S Mwebe, L Atwine, T Senkindu, T Natuhurira, C Katemba, E Ninsiima, M Acaku J Kyomuhangi, R Ankunda, D Tukwasibwe, L Ayesiga. University of Zimbabwe Clinical Research Centre, Harare, Zimbabwe: J Hakim, K Nathoo, M Bwakura-Dangarembizi, A Reid, E Chidziva, T Mhute, GC Tinago, J Bhiri, S Mudzingwa, M Phiri, J Steamer, R Nhema, C Warambwa, G Musoro, S Mutsai, B Nemasango, C Moyo, S Chitongo, K Rashirai, S Vhembo, B Mlambo, S Nkomani, B Ndemera, M Willard, C Berejena, Y Musodza, P Matiza, B Mudenge, V Guti. KEMRI Wellcome Trust Research Programme, Kilifi, Kenya: A Etyang, C Agutu, J Berkley, K Maitland, P Njuguna, S Mwaringa, T Etyang, K Awuondo, S Wale, J Shangala, J Kithunga, S Mwarumba, S Said Maitha, R Mutai, M Lozi Lewa, G Mwambingu, A Mwanzu, C Kalama, H Latham, J Shikuku, A Fondo, A Njogu, C Khadenge, B Mwakisha. Moi University Clinical Research Centre, Eldoret, Kenya: A Siika, K Wools-Kaloustian, W Nyandiko, P Cheruiyot, A Sudoi, S Wachira, B Meli, M Karoney, A Nzioka, M Tanui, M Mokaya, W Ekiru, C Mboya, D Mwimali, C Mengich, J Choge, W Injera, K Njenga, S Cherutich, M Anyango Orido,G Omondi Lwande, P Rutto, A Mudogo, I Kutto, A Shali, L Jaika, H Jerotich, M Pierre. Department of Medicine and Malawi-Liverpool Wellcome Trust Clinical Research Programme, College of Medicine, Blantyre, Malawi: J Mallewa, S Kaunda, J Van Oosterhout, B O’Hare, R Heydermann, C Gonzalez, N Dzabala, C Kelly, B Denis, G Selemani, L Nyondo Mipando, E Chirwa, P Banda, L Mvula, H Msuku, M Ziwoya, Y Manda, S Nicholas, C Masesa, T Mwalukomo, L Makhaza, I Sheha, J Bwanali, M Limbuni.

*Trial Coordination and Oversight*: MRC Clinical Trials Unit at UCL, London, UK: D Gibb, M Thomason, AS Walker, S Pett, A Szubert, A Griffiths, H Wilkes, C Rajapakse, M Spyer, A Prendergast, N Klein. Data Management Systems: M Rauchenberger, N Van Looy, E Little, K Fairbrother.

*Social Science Group*: F Cowan, J Seeley, S Bernays, R Kawuma, Z Mupambireyi.

Independent REALITY Trial Monitors: F Kyomuhendo, S Nakalanzi, J Peshu, S Ndaa, J Chabuka, N Mkandawire, L Matandika, C Kapuya.

*Trial Steering Committee*: I Weller (Chair), E Malianga, C Mwansambo, F Miiro, P Elyanu, E Bukusi, E Katabira, O Mugurungi, D Gibb, J Hakim, A Etyang, P Mugyenyi, J Mallewa.

*Data Monitoring Committee:* T Peto (Chair), P Musoke, J Matenga, S Phiri.

*Endpoint Review Committee* (independent members): H Lyall (Co-Chair), V Johnston (Co-Chair), F Fitzgerald, F Post, F Ssali, A Prendergast, A Arenas-Pinto, A Turkova, A Bamford.
