# Supplementary material for: Late Presentation With HIV in Africa: Phenotypes, Risk, and Risk Stratification in the REALITY Trial
Source: Clin Infect Dis. 2018 Mar 4;66(Suppl 2):S140–6. doi: 10.1093/cid/cix1142 (PMC5850547; doi:10.1093/cid/cix1142)
Supplement: Supplemental Data [file cix1142_suppl_supplemental_data.docx]

**SUPPLEMENTARY METHODS**

*Statistical analysis*

Baseline demographics, laboratory results, physical measurements and EQ-5D scores were all considered as predictors of mortality during the first 48 weeks on ART, after which participants exited the trial (Table 1, Supplementary Table 1). Illnesses and symptoms reported at enrolment were included if they occurred in >5% of participants; highly-related symptoms were identified through hierarchical clustering of all symptoms across patients and pallor (grouped with difficulty walking) and weakness, poor appetite and cough (grouped with weight loss) excluded. The mean of continuous factors measured at both screening and enrolment visits was used. Continuous factors that were highly correlated (Spearman rho>0.9) or missing in >10% were also excluded (namely body fat %, muscle mass, total body water, total body water %, bone mass, basal metabolic rate, CD3, CD8, lymphocytes, lymphocytes %, neutrophils %, MCV, red blood cells, haematocrit, phosphate, bilirubin, ALT and AST). Continuous factors with evidence of outliers were truncated at the 1^st^, 5^th^, 95^th^ or 99^th^ percentile based on the distribution. Previous healthcare contact was defined as having a chronic health condition (e.g. diabetes, hypertension) or having medication prescribed at least 14 days prior to the screening visit.

Factors were selected for the final Cox proportional hazards model using backwards elimination (exit p>0.1 to build an explanatory model, reporting only factors with p<0.05). Non-linearity was included in the model building process using multivariable fractional polynomials if p<0.05 (stata mfp). Clinical centre (reflecting clinical management and access to diagnostic facilities) was included in all models. Models were restricted to complete cases, so after initial variable selection a final model was refitted to all observations with complete data and the remaining factors were re-checked and included if p<0.1. All continuous factors were included in final models with linear effects, ie each unit increase had the same impact on mortality at all levels of the factor. The only interaction (p<0.01) between factors in this model was between albumin and fever (p=0.002). This suggested that albumin had little effect on mortality in the 13% of patients reporting fever at baseline (HR=1.00 per g/l higher (95% CI 0.97-1.04)) compared with higher albumin significantly reducing risk in the 87% without fever at baseline (HR=0.94 (0.91-0.96)). As the effects of other factors were similar including or excluding this interaction, it was not included in final models.

Different patterns of “late presenters” were identified using cluster analysis. First, principal components analysis of the correlation matrix was used to reduce the continuous factors to the smallest number of components that explained >75% of the variance. Hierarchical clustering then used Ward’s linkage on these principal components and remaining binary factors with the number of clusters determined using the Duda-Hart stopping rule.

**Supplementary Table 1 Other baseline factors not considered as predictors of mortality due to incompleteness, collinearity with other factors already being considered, or reflecting subsequent treatment**

|  | Died before 48 weeks  N=255 | Last seen alive  N=1580 | All participants  N=1805 |
| --- | --- | --- | --- |
| Temperature (˚C) (N=1793) | 36.7 (36.2, 37.3) | 36.5 (36.2, 36.9) | 36.5 (36.2, 36.9) |
| Height (cm) (N=1798) | 163 (157, 169) | 164 (158, 171) | 164 (158, 171) |
| Weight (kg) (N=1800) | 49.3 (42.1, 53.9) | 53.2, (46.8, 59.9) | 52.5 (46.3, 59.3) |
| CD3 (cells/mm^3^) (N=1734) | 431 (295, 691) | 584 (386, 843) | 564 (368, 828) |
| CD4% (N=1351) | 5 (3, 8) | 5 (3, 8) | 5 (3, 8) |
| CD8 (cells/mm^3^) (N=1693) | 394 (241, 615) | 501 (319, 748) | 482 (308, 726) |
| CD8% (N=1350) | 82 (62, 90) | 70 (56, 86) | 71 (57, 87) |
| CD4/CD8 ratio (N=1693) | 0.06 (0.03, 0.11) | 0.07 (0.04, 0.12) | 0.07 (0.04, 0.12) |
| Red blood cells (10^12^/l) (N=952) | 3.79 (3.18, 4.43) | 4.29 (3.71, 4.75) | 4.20 (3.64, 4.71) |
| Haemoglobin (g/dl) (N=1802) | 10.0 (8.4, 11.6) | 11.4 (9.7, 12.8) | 11.2 (9.6, 12.7) |
| Grade 1 or 2 | 74 (33%) | 359 (23%) | 433 (24%) |
| Grade 3 or 4 | 37 (16%) | 80 (5%) | 117 (6%) |
| White blood cells (10^9^/l) (N=1802) | 3.9 (2.9, 5.4) | 3.5 (2.7, 4.6) | 3.5 (2.7, 4.7) |
| Grade 1 or 2 | 39 (17%) | 303 (19%) | 342 (19%) |
| Grade 3 or 4 | 3 (1%) | 31 (2%) | 34 (2%) |
| Platelets (10^9^/l) (N=1801) | 260 (189, 341) | 251 (182, 343) | 252 (183, 343) |
| Grade 1 or 2 | 22 (10%) | 128 (8%) | 150 (8%) |
| Grade 3 or 4 | 1 (<1%) | 23 (1%) | 24 (1%) |
| Neutrophils (10^9^/l) (N=1785) | 1.99 (1.38, 3.07) | 1.69 (1.15, 2.50) | 1.72 (1.17, 2.58) |
| Grade 1 or 2 | 23 (10%) | 244 (15%) | 267 (15%) |
| Grade 3 or 4 | 8 (4%) | 33 (2%) | 41 (2%) |
| Lymphocytes (10^9^/l) (N=1792) | 0.86 (0.58, 1.20) | 0.98 (0.69, 1.39) | 0.96 (0.68, 1.35) |
| Grade 1 or 2 | 37 (17%) | 189 (12%) | 226 (13%) |
| Grade 3 or 4 | 36 (16%) | 143 (9%) | 179 (10%) |
| Lymphocytes % (N=1748) | 22.1 (14.2, 32.0) | 29.1 (21.2, 38.0) | 28.4 (20.0, 37.4) |
| Neutrophils % (N=1739) | 53.0 (41.7, 68.1) | 50.0 (39.3, 61.3) | 50.3 (39.6, 61.8) |
| Haematocrit (%) (N=695) | 30.0 (25.4, 35.3) | 34.5 (29.2, 38.8) | 33.9 (28.7, 38.4) |
| MCV (fl) (N=1694) | 83 (77, 88) | 84 (79, 89) | 84 (79, 89) |
| AST (U/I) (N=791) | 49 (33, 74) | 38 (28, 57) | 39 (29, 61) |
| Grade 1 or 2 | 52 (23%) | 199 (13%) | 251 (14%) |
| Grade 3 or 4 | 4 (2%) | 6 (<1%) | 10 (1%) |
| Creatinine (umol/l) (N=1796) | 76 (60, 97) | 71 (57, 86) | 71 (57, 87) |
| ALT (U/I) (N=1772) | 27 (16, 41) | 26 (17, 42) | 26 (17, 41) |
| Grade 1 or 2 | 35 (15%) | 270 (17%) | 305 (17%) |
| Grade 3 or 4 | 2 (1%) | 5 (<1%) | 7 (<1%) |
| Bilirubin (umol/l) (N=1767) | 6 (4, 10) | 6 (4, 9) | 6 (4, 9) |
| Grade 1 or 2 | 9 (4%) | 27 (2%) | 36 (2%) |
| Grade 3 or 4 | 2 (1%) | 5 (<1%) | 7 (<1%) |
| Phosphate (mg/dl) (N=1445) * | 2.06 (1.14, 3.58) | 1.45 (1.08, 3.20) | 1.49 (1.09, 3.26) |
| Malaria test done | 6 (3%) | 13 (1%) | 19 (1%) |
| Ultrasound performed | 3 (1%) | 7 (0.4%) | 10 (1%) |
| X ray performed | 6 (3%) | 10 (1%) | 16 (1%) |
| AFB performed | 3 (1%) | 7 (<1%) | 10 (1%) |
| Any investigation performed | 15 (7%) | 34 (2%) | 49 (3%) |
| Physician-reported conditions |  |  |  |
| Herpes simplex* |  |  |  |
| Current | 1 (<1%) | 9 (1%) | 10 (1%) |
| Previous | 1 (<1%) | 7 (<1%) | 8 (<1%) |
| Septicaemia* |  |  |  |
| Current | 0 | 1 (<1%) | 1 (<1%) |
| Previous | 1 (<1%) | 4 (<1%) | 5 (<1%) |
| Anaemia |  |  |  |
| Current | 6 (3%) | 13 (1%) | 19 (1%) |
| Previous | 1 (<1%) | 11 (1%) | 12 (1%) |
| Chronic diarrhoea* |  |  |  |
| Current | 3 (1%) | 45 (3%) | 48 (3%) |
| Previous | 11 (5%) | 75 (5%) | 86 (5%) |
| Kaposi’s sarcoma |  |  |  |
| Current | 3 (1%) | 11 (1%) | 14 (1%) |
| Abscess * |  |  |  |
| Current | 1 (<1%) | 2 (<1%) | 3 (<1%) |
| Previous | 1 (<1%) | 5 (<1%) | 6 (<1%) |
| Meningitis* |  |  |  |
| Current | 3 (1%) | 1 (<1%) | 4 (<1%) |
| Previous | 1 (<1%) | 3 (<1%) | 4 (<1%) |
| Pneumonia* |  |  |  |
| Current | 10 (4%) | 26 (2%) | 36 (2%) |
| Previous | 11 (5%) | 62 (4%) | 73 (4%) |
| Cryptococcus* |  |  |  |
| Current | 4 (2%) | 17 (1%) | 21 (1%) |
| Previous | 2 (1%) | 1 (<1%) | 3 (<1%) |
| Chronic fever* |  |  |  |
| Current | 10 (4%) | 14 (1%) | 24 (1%) |
| Previous | 7 (3%) | 9 (<1%) | 16 (<1%) |
| Other infection* |  |  |  |
| Current | 1 (<1%) | 5 (<1%) | 6 (<1%) |
| Previous | 1 (<1%) | 8 (1%) | 9 (1%) |
| Any infection (* above, or TB or WHO 3/4 candida) |  |  |  |
| Current | 79 (35%) | 382 (24%) | 461 (26%) |
| Previous | 34 (15%) | 234 (15%) | 268 (15%) |
| Previous wasting/severe weight loss (WHO 3/4) | 19 (8%) | 63 (4%) | 82 (5%) |
| Previous TB (all) (WHO 3/4) | 6 (3%) | 37 (2%) | 43 (2%) |
| Previous candida (WHO 3/4) | 25 (11%) | 133 (8%) | 158 (9%) |
| Patient reported symptoms |  |  |  |
| Dehydration | 36 (16%) | 59 (4%) | 95 (5%) |
| Ear discharge | 8 (4%) | 22 (1%) | 30 (2%) |
| Jaundice | 5 (2%) | 16 (1%) | 21 (1%) |
| Visual problems | 17 (8%) | 48 (3%) | 65 (4%) |
| Bruises | 8 (4%) | 28 (2%) | 36 (2%) |
| Poor sleep | 32 (14%) | 105 (7%) | 137 (8%) |
| Muscle ache | 37 (16%) | 124 (8%) | 161 (9%) |
| Headache | 15 (7%) | 49 (3%) | 64 (4%) |
| Difficulty breathing | 21 (9%) | 39 (2%) | 60 (3%) |
| Diarrhoea | 21 (9%) | 93 (6%) | 114 (6%) |
| Weakness | 151 (67%) | 526 (33%) | 677 (38%) |
| Pallor | 71 (32%) | 154 (10%) | 225 (12%) |
| Poor appetite/difficulty feeding | 122 (54%) | 458 (29%) | 580 (32%) |
| Cough | 82 (36%) | 275 (17%) | 357 (20%) |
| Depression | 35 (16%) | 120 (8%) | 155 (9%) |
| Body fat % (N=1743) | 9.8 (4.6, 19.0) | 13.1 (8.0, 22.8) | 12.8 (7.7, 22.6) |
| Lean (muscle) mass (kg) (N=1740) | 38.8 (35.3, 44.7) | 41.6 (37.4, 47.2) | 41.3 (37.0, 46.8) |
| Total body water (kg) (N=1731) | 29.8 (25.9, 34.4) | 31.6 (27.4, 35.4) | 31.3 (27.3, 35.3) |
| Total body water % (N=1731) | 63.6 (56.4, 70.1) | 60.3 (53.3, 65.6) | 60.7 (53.5, 65.9) |
| Bone mass (kg) (N=1675) | 2.1 (1.9, 2.4) | 2.3 (2.0, 2.5) | 2.3 (2.0, 2.5) |
| Basal metabolic rate (kcal) (N=1674) | 1218 (1128, 1371) | 1312 (1201, 1450) | 1303 (1192, 1441) |
| Treatment |  |  |  |
| Started ART with efavirenz | 203 (90%) | 1416 (90%) | 1619 (90%) |
| Started ART with tenofovir/ emtricitabine | 170 (76%) | 1252 (79%) | 1422 (79%) |
| Initial ART regimen (excluding raltegravir) |  |  |  |
| Tenofovir/emtricitabine/efavirenz | 164 (73%) | 1214 (77%) | 1378 (76%) |
| Zidovudine/lamivudine/efavirenz | 29 (13%) | 150 (10%) | 179 (10%) |
| Zidovudine/lamivudine/nevirapine | 15 (7%) | 129 (8%) | 144 (8%) |
| Abacavir/lamivudine/efavirenz | 10 (4%) | 45 (3%) | 55 (3%) |
| Tenofovir/emtricitabine/nevirapine | 6 (3%) | 32 (2%) | 38 (2%) |
| Other | 1 (<1%) | 10 (1%) | 11 (1%) |
| Receiving isoniazid pre-randomization | 29 (13%) | 148 (9%) | 177 (10%) |
| Receiving isoniazid at randomization |  |  |  |
| Treatment | 31 (14%) | 191 (12%) | 222 (12%) |
| Prophylaxis | 83 (37%) | 704 (45%) | 787 (44%) |
| Receiving fluconazole pre-randomization | 40 (18%) | 165 (10%) | 205 (11%) |
| Receiving fluconazole at randomization |  |  |  |
| Treatment | 31 (14%) | 117 (7%) | 148 (8%) |
| Prophylaxis | 89 (40%) | 772 (49%) | 861 (48%) |
| Receiving azithromycin at randomization |  |  |  |
| Treatment | 2 (1%) | 11 (1%) | 13 (1%) |
| Prophylaxis | 98 (44%) | 805 (51%) | 903 (50%) |
| Receiving albendazole at randomization |  |  |  |
| Treatment | 0 | 4 (0.3%) | 4 (0.2%) |
| Prophylaxis | 98 (44%) | 806 (51%) | 904 (50%) |
| Receiving cotrimoxazole/dapsone at randomization |  |  |  |
| Cotrimoxazole: prophylaxis | 221 (98%) | 1545 (98%) | 1766 (98%) |
| Cotrimoxazole: treatment | 1 (0.4%) | 3 (0.2%) | 4 (0.2%) |
| Dapsone: prophylaxis | 3 (1%) | 29 (2%) | 32 (2%) |
| Receiving other antibiotics at randomization | 44 (19%) | 154 (10%) | 198 (11%) |
| Number of non-ART drugs receiving at randomization | 3 (2,4) | 3 (2,4) | 3 (2,4) |
| 1 | 30 (13%) | 266 (17%) | 296 (16%) |
| 2 | 52 (22%) | 330 (21%) | 378 (21%) |
| 3 | 41 (19%) | 353 (23%) | 401 (22%) |
| 4 | 58 (26%) | 360 (23%) | 418 (23%) |
| 5 | 19 (8%) | 145 (9%) | 164 (9%) |
| 6 | 9 (4%) | 70 (4%) | 79 (4%) |
| 7+ | 16 (7%) | 46 (3%) | 62 (3%) |

* counted as any infection.

**Supplementary Table 2 Characteristics of different groups of late presenters**

|  | Group 1 | Group 2 | Group 3 | Group 4 | Group 5 | Total |
| --- | --- | --- | --- | --- | --- | --- |
|  | N=355 | N=394 | N=242 | N=218 | N=398 | 1607 |
| **Died** | **87 (25%)** | **45 (11%)** | **25 (10%)** | **12 (6%)** | **15 (4%)** | **184 (11%)** |
| **Died by 12 weeks** | **65 (18%)** | **28 (7%)** | **12 (5%)** | **7 (3%)** | **5 (1%)** | **117 (7%)** |
| Male | 194 (55%) | 255 (65%) | 58 (24%) | 6 (3%) | 343 (86%) | 856 (53%) |
| **Age (years)** | **36 (29, 41)** | **35 (30, 42)** | **30 (23, 38)** | **36 (29, 41)** | **39 (33, 45)** | **36 (29, 42)** |
| Ever smoked | 79 (22%) | 99 (25%) | 11 (5%) | 4 (2%) | 112 (28%) | 305 (19%) |
| WHO stage |  |  |  |  |  |  |
| 1 | 51 (14%) | 53 (13%) | 30 (12%) | 32 (15%) | 83 (21%) | 249 (15%) |
| 2 | 47 (13%) | 89 (23%) | 82 (34%) | 117 (54%) | 165 (41%) | 500 (31%) |
| 3 | 154 (43%) | 199 (51%) | 104 (43%) | 54 (25%) | 123 (31%) | 634 (39%) |
| 4 | 103 (29%) | 53 (13%) | 26 (11%) | 15 (7%) | 27 (7%) | 224 (14%) |
| Hospitalized at randomization | 12 (3%) | 2 (1%) | 0 (0%) | 0 (0%) | 0 (0%) | 14 (1%) |
| Previous healthcare contact | 49 (14%) | 27 (7%) | 21 (9%) | 8 (4%) | 33 (8%) | 138 (9%) |
| **WHO grade 3/4 weight loss (current)** | **110 (31%)** | **93 (24%)** | **37 (15%)** | **17 (8%)** | **39 (10%)** | **296 (18%)** |
| TB (any; current) | 79 (22%) | 80 (20%) | 25 (10%) | 11 (5%) | 46 (12%) | 241 (15%) |
| WHO grade 3/4 candida (current) | 28 (8%) | 25 (6%) | 17 (7%) | 11 (5%) | 8 (2%) | 89 (6%) |
| **Fever** | **100 (28%)** | **48 (12%)** | **19 (8%)** | **20 (9%)** | **26 (7%)** | **213 (13%)** |
| Weight loss (any; patient reported) | 253 (71%) | 233 (59%) | 118 (49%) | 82 (38%) | 139 (35%) | 825 (51%) |
| Difficulty walking | 154 (43%) | 14 (4%) | 4 (2%) | 8 (4%) | 5 (1%) | 185 (12%) |
| Rash | 57 (16%) | 63 (16%) | 62 (26%) | 49 (22%) | 70 (18%) | 301 (19%) |
| Numbness | 135 (38%) | 76 (19%) | 29 (12%) | 32 (15%) | 40 (10%) | 312 (19%) |
| Abdominal ache | 71 (20%) | 40 (10%) | 18 (7%) | 26 (12%) | 17 (4%) | 172 (11%) |
| Sore mouth/ulcers/candida | 57 (16%) | 42 (11%) | 25 (10%) | 18 (8%) | 20 (5%) | 162 (10%) |
| **Vomiting** | **48 (14%)** | **21 (5%)** | **4 (2%)** | **11 (5%)** | **9 (2%)** | **93 (6%)** |
| **EQ5D: mobility** |  |  |  |  |  |  |
| **No problems** | **79 (22%)** | **374 (95%)** | **237 (98%)** | **202 (93%)** | **387 (97%)** | **1279 (80%)** |
| **Some problems** | **265 (75%)** | **20 (5%)** | **5 (2%)** | **16 (7%)** | **11 (3%)** | **317 (20%)** |
| **Confined to bed** | **11 (3%)** | **0 (0%)** | **0 (0%)** | **0 (0%)** | **0 (0%)** | **11 (1%)** |
| **EQ5D: self-care** |  |  |  |  |  |  |
| **No problems** | **102 (29%)** | **378 (96%)** | **239 (99%)** | **203 (93%)** | **389 (98%)** | **1311 (82%)** |
| **Some problems** | **203 (57%)** | **15 (4%)** | **3 (1%)** | **15 (7%)** | **9 (2%)** | **245 (15%)** |
| **Unable** | **50 (14%)** | **1 (0%)** | **0 (0%)** | **0 (0%)** | **0 (0%)** | **51 (3%)** |
| EQ5D: usual activities |  |  |  |  |  |  |
| No problems | 25 (7%) | 332 (84%) | 232 (96%) | 185 (85%) | 372 (93%) | 1146 (71%) |
| Some problems | 229 (65%) | 60 (15%) | 10 (4%) | 30 (14%) | 26 (7%) | 355 (22%) |
| Unable | 101 (28%) | 2 (1%) | 0 (0%) | 3 (1%) | 0 (0%) | 106 (7%) |
| EQ5D: pain |  |  |  |  |  |  |
| None | 33 (9%) | 294 (75%) | 204 (84%) | 152 (70%) | 330 (83%) | 1013 (63%) |
| Moderate | 290 (82%) | 97 (25%) | 36 (15%) | 63 (29%) | 66 (17%) | 552 (34%) |
| Extreme | 32 (9%) | 3 (1%) | 2 (1%) | 3 (1%) | 2 (1%) | 42 (3%) |
| EQ5D: anxiety |  |  |  |  |  |  |
| Not anxious | 134 (38%) | 320 (81%) | 228 (94%) | 163 (75%) | 345 (87%) | 1190 (74%) |
| Moderately | 202 (57%) | 69 (18%) | 13 (5%) | 48 (22%) | 50 (13%) | 382 (24%) |
| Extremely | 19 (5%) | 5 (1%) | 1 (0%) | 7 (3%) | 3 (1%) | 35 (2%) |
| Blood pressure (systolic/diastolic mmHg) | 100/65 (90/60, 110/72) | 104/70 (98/62, 110/73) | 107/70 (100/64, 116/76) | 110/70 (100/66, 119/77) | 119/75 (110/70, 130/85) | 109/70 (100/63, 118/77) |
| Fat mass (kg) | 4.2 (1.7, 7.5) | 4.4 (2.9, 7.4) | 7.1 (4.0, 11.3) | 18.9 (14.4, 24.7) | 7.8 (5.4, 12.5) | 6.6 (3.7, 12.3) |
| Fat free mass (kg) | 42.0 (37.8, 47.4) | 43.7 (40.0, 48.4) | 38.1 (36.0, 40.9) | 42.2 (40.2, 44.7) | 50.6 (46.5, 55.1) | 43.6 (39.2, 49.5) |
| MUAC (cm) | 22.3 (20.4, 24.4) | 22.8 (21.4, 24.1) | 22.6 (21.0, 24.0) | 28.0 (26.0, 30.1) | 25.6 (24.0, 27.7) | 24.0 (22.0, 26.1) |
| BMI (kg/m^2^) | 17.5 (16.0, 19.7) | 18.0 (16.8, 19.3) | 18.2 (16.8, 19.7) | 23.9 (22.0, 26.3) | 20.5 (19.0, 22.8) | 19.2 (17.3, 21.4) |
| **Grip strength (kg)** | **20.0 (15.0, 26.9)** | **24.5 (20.8, 30.5)** | **20.3 (16.8, 23.8)** | **23.6 (20.3, 27.2)** | **33.5 (27.8, 38.0)** | **24.5 (19.4, 31.1)** |
| **CD4 (cells/mm^3^)** | **28 (13, 49)** | **43 (20, 69)** | **27 (12, 48)** | **48 (24, 73)** | **42 (18, 67)** | **37 (17, 63)** |
| **Haemoglobin (g/dl)** | **10.1 (8.7, 11.8)** | **10.3 (9.1, 12.0)** | **10.7 (9.3, 12.1)** | **11.4 (10.5, 12.5)** | **12.8 (11.5, 14.1)** | **11.2 (9.6, 12.7)** |
| White blood cells (10^9^/l) | 3.5 (2.5, 4.8) | 4.4 (3.5, 5.7) | 3.0 (2.3, 4.0) | 3.2 (2.5, 4.0) | 3.4 (2.6, 4.3) | 3.5 (2.7, 4.7) |
| Platelets (10^9^/l) | 247 (177, 329) | 307 (223, 413) | 266 (184, 370) | 240 (186, 325) | 225 (167, 291) | 255 (184, 345) |
| Neutrophils (10^9^/l) | 1.71 (1.07, 2.68) | 2.49 (1.77, 3.56) | 1.42 (0.95, 1.93) | 1.54 (1.15, 2.10) | 1.59 (1.09, 2.18) | 1.74 (1.19, 2.58) |
| **Albumin (g/l)** | **32 (26, 37)** | **32 (28, 36)** | **37 (32, 41)** | **38 (34, 42)** | **39 (35, 42)** | **35 (30, 40)** |
| eGFR (ml/min) | 87.2 (67.6, 108.5) | 95.8 (76.5, 115.4) | 105.8 (85.0, 137.5) | 107.5 (88.8, 136.9) | 95.6 (78.4, 119.5) | 97.1 (77.5, 120.9) |
| Log10 HIV viral load | 5.6 (5.2, 6.0) | 5.4 (5.0, 5.8) | 5.3 (5.0, 5.7) | 5.2 (4.9, 5.6) | 5.4 (4.9, 5.7) | 5.4 (5.0, 5.8) |
| *Not included in the cluster analysis* |  |  |  |  |  |  |
| CD8 (cells/mm^3^) | 408 (257, 643) | 528 (352, 801) | 446 (303, 764) | 523 (355, 700) | 499 (335, 719) | 479 (310, 724) |
| Phosphate (mg/dl) | 2.72 (1.25, 3.67) | 1.30 (1.01, 2.24) | 1.43 (1.14, 3.00) | 1.39 (1.01, 3.23) | 1.39 (1.04, 3.06) | 1.44 (1.06, 3.20) |
| Diarrhoea | 32 (9%) | 22 (6%) | 13 (5%) | 12 (6%) | 20 (5%) | 99 (6%) |
| Poor appetite/difficulty feeding | 212 (60%) | 115 (29%) | 60 (25%) | 59 (27%) | 66 (16%) | 512 (32%) |
| Dehydration | 55 (15%) | 14 (4%) | 3 (1%) | 1 (<1%) | 2 (1%) | 75 (5%) |

Note: factors in bold have p<0.05 in the final multivariable model for mortality.
